# The metabolic pathways and environmental controls of hydrocarbon biodegradation in marine ecosystems

**DOI:** 10.3389/fmicb.2014.00471

**Published:** 2014-09-04

**Authors:** Joel E. Kostka, Andreas P. Teske, Samantha B. Joye, Ian M. Head

**Affiliations:** ^1^School of Biology and Earth and Atmospheric Sciences, Georgia Institute of TechnologyAtlanta, GA, USA; ^2^Department of Marine Sciences, University of North CarolinaChapel Hill, NC, USA; ^3^Department of Marine Sciences, University of GeorgiaAthens, GA, USA; ^4^School of Civil Engineering and Geosciences, Newcastle UniversityNewcastle upon Tyne, UK

**Keywords:** Deepwater Horizon, biodegradation, Gulf of Mexico, hydrocarbon, oil spill, bacteria, microbial communities, Metagenomics

Hydrocarbon-degrading microorganisms are ubiquitous in the world's oceans (Head et al., 2006; Yakimov et al., 2007), and biodegradation mediated by indigenous microbial communities is the ultimate fate of the majority of oil hydrocarbon that enters the marine environment (Leahy and Colwell, 1990; Prince, 2010; Atlas and Hazen, 2011). In response to the natural complexity of hydrocarbon compounds found in petroleum deposits, diverse marine microorganisms have evolved with an equal complexity of metabolic pathways to take advantage of hydrocarbons as a rich carbon and energy source. To minimize the environmental impact of oil spills and to optimize the environmental benefits of biodegradation, it is essential to uncover the metabolic potential of hydrocarbon-degrading bacteria and to address the factors that limit microbially-catalyzed biodegradation *in situ*.

Microbial community structure and diversity likely defines the metabolic potential of oil-degrading communities (Head et al., 2006). Thus, it is critical to understand the relationships between microbial community structure and the metabolic activity of hydrocarbon-degrading microbial groups. Much progress has been made to determine the response of specific microbial taxa to oil discharge in marine environments impacted by oil spills or natural seeps. However, the majority of studies of hydrocarbon-degrading microorganisms have been conducted in laboratory cultures, and our ability to understand and predict the dynamics of *in situ* microbial communities responding to environmental stimuli such as the presence of oil hydrocarbons remains in its infancy (Prosser et al., 2007).

*In situ* characterization of hydrocarbon-degrading microbial communities was hampered until recently by practical limitations of molecular biology techniques in phylogenetic resolution and depth of coverage (Gilbert and Dupont, 2011; Jansson et al., 2012). Advances in next generation sequencing technologies and the use of stable isotope tracers have greatly improved our ability to interrogate the phylogenetic and functional diversity of hydrocarbon-degrading microorganisms in the field. The development and application of omics approaches have led to the characterization of novel biochemical pathways of biogeochemical significance. This Research Topic focuses on investigations that utilize the latest molecular and biogeochemical techniques, (including high throughput sequencing, isotope tracers, and omic approaches) to render a predictive understanding of the biogeochemical processes and metabolic pathways that in turn regulate the impacts and biodegradation of petroleum hydrocarbons released into the marine environment.

The Deepwater Horizon (DWH) blowout that occurred in the Gulf of Mexico in 2010 is distinguished as the largest accidental marine oil spill in history (Atlas and Hazen, 2011), and the DWH spill represents the first major event in which next-generation sequencing approaches have been applied to illustrate with high resolution the dramatic changes in the abundance, structure, and metabolic potential of microbial communities in oil-impacted marine ecosystems (Joye et al., 2014; King et al., 2014). In the first 8 articles of this Research Topic, the latest microbiological and biogeochemical approaches are employed to interrogate the diversity, metabolic potential, and environmental forcings of hydrocarbon-degrading microbial communities in response to oil discharged during the DWH blowout. Smith et al. (2013) provide insight into the potential for alkane degradation by prespill or indigenous bacterioplankton in the northern Gulf of Mexico using high-throughput analysis of genes encoding alkane hydroxylase, alkB, one of the best known molecular marker genes for hydrocarbon degradation. In Mason et al. (2014), the metabolic potential of *Colwellia*, a bacterium detected in high abundance in Gulf waters impacted by the DWH spill, is determined using single-cell genomics. A series of papers then describes the impacts of Macondo oil (released from the DWH discharge) on the community structure and metabolic function of benthic microbial communities using omics techniques (Kappell et al., 2014; Lamendella et al., 2014; Scott et al., 2014; Thomas et al., 2014). Biodegradation and the impacts of Macondo oil are then assessed using geochemical methods in studies of the nearshore water column (Ziervogel et al., 2014) and sediments (Elango et al., 2014).

A major theme of this special issue is to bridge laboratory-based studies of biodegradation to those conducted in the field. Cravo-Laureau and Duran (2014) review how mesocosm experiments have been used to dissect hydrocarbon degradation mechanisms by somewhat reducing environmental complexity and serving as a transition between the lab and the field. Röling and van Bodegom (2014) further posit how observations of oil degradation potential at the cellular level could be scaled to the community as well as ecosystem level using systems biology and omics-based approaches.

Although biodegradation was shown to be successful in naturally remediating oil contamination associated with several spills that impacted marine shorelines (Head et al., 2006; Prince, 2010; Atlas and Hazen, 2011), much remains to be learned about the environmental controls of hydrocarbon degradation in marine sediments. Five papers in this issue evaluate the environmental conditions (oxygen availability, nutrient levels, oil chemistry) controlling biodegradation in sediments with experimental and modeling approaches. Under aerobic conditions, Singh et al. (2014) study the kinetic parameters of crude oil-degrading microbial communities in response to nutrient and oil loading in beach sand microcosms. High nutrient levels are shown to select for members of the hydrocarbonoclastic genus, *Alcanivorax*, while selecting against aromatic-degrading *Cycloclasticus* sp. Bose et al., 2013) quantify oxidation rates of short chain alkanes under sulfate-reducing conditions and explore the use of stable C isotopes to trace biodegradation activity in microcosms of cold seep sediments. Sherry et al. (2014) show that the composition of the crude oil itself may play a critical role as volatile hydrocarbons inhibit biodegradation under methanogenic conditions. Capping and *in situ* aeration are shown to effectively remediate and detoxify buried oil in anaerobic marine sediments by Genovese et al. (2014). Torlapati and Boufadel (2014) present a numerical model that employs genetic algorithms to predict biodegradation kinetics for oil entrapped in sediments.

Finally, two papers in this issue explore the mechanisms of hydrocarbon degradation using novel cultivation methods under aerobic and anaerobic conditions. Mishamandani et al. (2014) use stable isotope probing to reveal that the aerobic methylotroph, *Methylophaga*, is capable of growth on alkanes as the sole source of carbon and energy. Lyles et al. (2014) investigate the linkages between the metabolism of hydrocarbon-degrading syntrophs and steel corrosion in electrochemical cells designed to simulate oil production systems.

By closely coupling cutting-edge microbiological (omics) and biogeochemical (stable isotope tracers) methods, the dynamics and selection of microbial populations responding to the chemical evolution of oil hydrocarbons has just begun to be revealed in marine ecosystems. In general, observations made from studies carried out before the advent of next generation sequencing technologies have been supported by recent work. Moreover, the challenge remains to definitively link the structure and function of hydrocarbon-degrading microbial groups to improve predictive models of biodegradation.

## Conflict of interest statement

The authors declare that the research was conducted in the absence of any commercial or financial relationships that could be construed as a potential conflict of interest.
